# Family members’ experiences of everyday caregiving for a family member living with Parkinson’s disease: a qualitative thematic analysis study

**DOI:** 10.1186/s12912-024-01767-6

**Published:** 2024-02-06

**Authors:** Zvonka Fekonja, Nadja Irgolič, Dominika Vrbnjak

**Affiliations:** 1 Faculty of Health Sciences, University of Maribor, Žitna Ulica 15, 2000 Maribor, SI Slovenia; 2 Dom Danice Vogrinec Maribor, Unit Tabor, Čufarjeva Cesta 9, 2000 Maribor, SI Slovenia

**Keywords:** Caregivers, Parkinson’s disease, Family, Patients, Experiences, Patient care

## Abstract

**Background:**

In the daily life of individuals living with Parkinson's disease, their loved ones are crucial. Adapting family members to the patient's condition, support in providing care, and psychosocial adaptations is essential.

**Aim:**

To explore family members' perception of everyday caregiving for a family member living with Parkinson's disease and to describe their role in the care and everyday life.

**Methods:**

In a descriptive, qualitative thematic analysis study, semi-structured interviews were conducted with ten people between the ages of 20 and 70, the closest family members of people living with Parkinson's disease. The analysis of the collected data was carried out using thematic analysis.

**Results:**

We generated the main theme: "Living with a family member with Parkinson’s disease", with associated secondary-level sub-themes: “Response”, “Change”, “Care”, and “Support”. Family members of individuals living with Parkinson's disease frequently encounter similar life situations. The most notable transformation in their daily lives primarily revolves around adapting to various activities.

**Conclusions:**

Family members are the ones who most often take on the role of caregiver and provide help to their loved ones. Many of them accept the disease as a part of everyday life and learn to live with it. It is of fundamental importance that we offer family members the necessary support, knowledge, and involvement in holistic treatment and care.

**Supplementary Information:**

The online version contains supplementary material available at 10.1186/s12912-024-01767-6.

## Introduction

The increase in life expectancy over the past few decades has resulted in a rise in chronic diseases, including Parkinson's disease. Parkinson's disease is a multi-systemic neurodegenerative disorder characterized by motor impairments such as tremor, rigidity, bradykinesia, akinesia, and balance disorders, as well as non-motor impairments such as depression, sleep disturbances, and pain [1]. Parkinson's disease is age-related [2] and typically manifests between the ages of 50 and 65 [3]. It is the second most common neurodegenerative disease after Alzheimer’s [4], affecting 1% of people over 60 in developed countries [3]. Approximately 10 million people worldwide are living with the disease. Prevalence is increasing, and it is projected that the number of Parkinson’s patients will double by 2030 [5].

The effectiveness of pharmacological treatment gradually decreases as the disease progresses due to the progressive degeneration and disruption of dopamine production in the brain [6]. For many patients, Parkinson's disease medications remain effective for only around ten years [7]. While techniques like deep brain stimulation have shown promise in improving the condition, awareness and access to such treatments are still limited [8]. The use of deep brain stimulation also involves providing psychosocial support to help family members adapt to the ongoing challenges of daily life [9]. As a result, many individuals live with Parkinson's disease for the rest of their lives and, as the disease progresses, become increasingly disabled until they eventually become immobile [10]. Following a progressive decline in functional abilities, the disorder renders the individual incapable of self-care, resulting in complete dependence. Often, family members initially provide care, assistance, and support to individuals living with Parkinson's disease [11].

Parkinson's disease has an impact on the individuals, the entire family and the broader circle of friends and loved ones, as the responsibility and burden of caring for an individual living with Parkinson's disease falls mainly on them [12]. In recent literature, an informal carer refers to an individual who provides physical and psychological care to a person in need [13]. Most often, the patient's close family members, such as spouses or adult children, are the ones primarily affected [14]. The motivation of family members to care for the ill individual is related to expressions of care, love, gratitude, or respect for the family member [15].

Padovani et al. [16] state that the partner or spouse most often assumes the role of primary caregiver, not only due to emotional closeness but also because of the sense of responsibility inherent in marriage and adherence to socio-cultural norms and values. However, researchers [16, 17] find that family members often report feelings of sadness and depression as the most prevalent emotions. These feelings become apparent when an affected family member is faced with the limitations imposed by the disease, instilling fear in their loved ones as well. Nevertheless, family members, when progressive disease occurs, often do not receive sufficient information and support, especially regarding the prognosis and what to expect from the disease’s progression [18]. Usually, family members and individuals living with Parkinson’s’ disease feel unprepared for the challenges and experience distress [19].

Research indicates that Parkinson's disease affects individuals and those close to them, having a significant impact on their daily lives [20]. Factors such as coping strategies, personality, values, beliefs, and life experiences directly influence the process of psychosocial adjustment [21]. Psychosocial adjustment is a complex, dynamic, cyclical and interactive process that significantly affects the quality of life of the patient and their family members [22]. Årestedt et al. [23], argue that healthcare and treatment interventions should focus on patients and their family members to achieve better outcomes regarding self-care, acceptance of the condition, and psychosocial adjustment. Caring for individuals with complex chronic illnesses like Parkinson's affects family members, as caring responsibilities can affect financial stability as well as physical, social and emotional health and well-being [19]. It is important that family members adapt to the person's condition, provide support in the provision of care and make psychosocial adjustments [24]. Without flexible coping mechanisms and support from other family members and society, the condition can lead to declining health and quality of life for family members [8]. Recent literature highlights the need for caregiver-specific interventions in Parkinson's disease. Sturm et al. [25] note that many interventions are integrated into patient treatments rather than targeting caregivers. Mosley et al. [5] suggest strategies like educational support, psychotherapy for caregivers, and managing patients' neuropsychiatric symptoms. Geerlings et al. [26] emphasize customizing these interventions for diverse caregiver needs. It is crucial to find out how to provide support, guidance, and psychosocial stability to the family in caregiving for a family member living with Parkinson’s disease. The initial step is to understand family members' experiences, roles, and primary concerns that contribute to providing insights for developing effective, personalized caregiver support. Therefore, the study aimed to explore the perceptions of family members regarding everyday caregiving for a family member living with Parkinson's disease and to describe their role in care and everyday life.

## Methods

### Study design

We used a descriptive qualitative thematic analysis study [27] to understand better how family members cope with daily care for a loved one with Parkinson's disease. The consolidated qualitative research (COREQ) reporting criteria was utilized [28].

### Setting and participants

The second author approached members of the society of patients with Parkinsonism and other extrapyramidal disorders in Slovenia. Purposive sampling was utilized for participant selection. In total, 10 family members were interviewed. The sample consisted of the closest family members of Parkinson's patients who lived with them, including spouses and adult children, who volunteered to participate in the study. No participant refused to participate in the study. Data saturation was attained after conducting ten interviews. The participants' ages ranged between 20 and 70. Seven of them were spouses of older people, and three were children of family members living with Parkinson’s disease.

### Data collection and analysis

Interviews were conducted between September and November 2022 by the second author (MSc student, female) under the supervision of two experienced researchers in the nursing field (PhD-prepared females). The family members were recruited by personal invitation with the help of the society of patients with Parkinsonism and other extrapyramidal disorders in Slovenia. The second author arranged the interview's date and time according to the family members’ preferences. Interviews took place on the premises of the society. The interview consisted of ten semi-structured questions, including questions about socio-demographic characteristics. The interviews were conducted using an interview guide that included questions pertinent to the research objectives (Suppl. 1). Examples of the questions asked included: "How would you describe life with a Parkinson's patient?"; "What is the biggest burden for you in your life with a Parkinson's patient?"; and "How has your relationship with the patient changed compared to before the illness? Where do you notice the most significant changes? How does the disease impact your relationships with other family members and friends?" To ensure narrative responses probing questions included words like "how," "why," or "can you describe," after each question. The interviews took place in a calm environment chosen by the participants. The interview lasted between 25 and 40 min. They were recorded and transcribed verbatim for analysis. Data collection was a one-time event at a place chosen by the interviewee that was suitable for the interview. There were no plans for further contact with the research participants.

We used the six steps of the inductive thematic analysis process by Braun and Clarke [27]. Experienced and trained researchers in qualitative studies engaged with data independently. This process included in-depth readings, re-readings of the transcript, highlighting critical information, note-taking, and initial code generation. Next, researchers examined the codes, identifying how they fit together to construct common themes. Frequent team meetings facilitated the collective generation and review of themes, intending to reach a consensus for the final refinement of themes through comprehensive discussions. The true perceptions of Parkinson's patients' family members were captured through direct quotations from the primary data. MAXQDA Analytics Pro 2022 was used to support data management.

### Trustworthiness

Lincoln and Guba's [29] criteria for trustworthiness were followed: credibility, dependability, confirmability and transferability. Credibility was achieved by transcribing the recorded interviews verbatim without adding or subtracting text. Also, two researchers analyzed data independently. To ensure dependability, the entire sampling process, data collection and analysis was fully documented rigorously and consistently. Confirmability was ensured by continuous review and refinement of data during collection and analysis to ascertain that the findings could be replicated by other researchers. Direct quotations of the family members support the interpretation of the findings. To achieve transferability, the authors provided a detailed account of the study's setting, family member’s profiles, sampling techniques, and data collection location. This thorough explanation aimed to enhance the reader's confidence in the applicability of the data to other contexts. Additionally, an ethics expert and the research team reviewed and revised the interview guide before data collection, further reinforcing the study's integrity.

### Ethical considerations

The research study obtained approval from the relevant ethics commission (038/2022/5475–5/902). Study participants were provided a written explanation detailing the research's aim and objectives. It was explicitly stated that participation in the study was voluntary, ensuring the preservation of anonymity and confidentiality. Participants were also informed of their right to withdraw from the study at any stage. The identities of all interviewees were concealed and replaced with pseudonyms to ensure anonymity.

## Results

The inductive thematic analysis led to the generation of the main theme: “Living with a family member with Parkinson’s disease”, and four secondary level sub-themes: (1) Response; (2) Change; (3) Care; and (4) Support (Table 1).

**Table 1:** Summary of thematic analysis

**Main themes**	**Secondary subthemes**	**Primary subthemes**	**Free codes**
**Living with a family member with Parkinson’s disease**	Response	Expression of Emotions	Fear, confusion, worry, anger, sadness, loneliness, distress, helplessness, anxiety, unpleasantness, patience, motivation, will, effort, bad conscience, shock, resistance, disconnection, pain, neglect, denial, perseverance, humility, trust
		Adaptation	Daily learning, new skills, coping with difficult situations, appreciation of what is good/healthy, accepting help, giving help, more calmness in life
	Change	Role of family members	Adjustment to daily life, change in lifestyle, different activities than before, change in behaviour, increased responsibility, getting used to a different life, social isolation, inevitability, reduced quality of life, reduced activities
		Patient characteristics	Dependence on help from others, inability to care for self, increased sports activity, changes in mobility, slowness, quick to take offence, self-absorption, resentfulness, intense emotions, sensitivity, listlessness, unmotivated, disinterested, denial, unpreoccupied, irritable
		Family relationships	Same as before illness, more connected, closer, socially isolated, relationship problems, otherness, relationship deterioration, marital problems, relationship change
	Care	Care and assistance	Care and assistance with activities of daily living, assistance with personal care, assistance with outside activities, assistance with household chores, involvement in medical treatment, encouragement to walk, service
		Stress and burden	Stress, giving up, extra workload, unpredictability, disease progression, lack of awareness, complications of the disease, side effects of medication
	Support	Society	Association, training, seminars, workshops, lectures, socialising, games, excursions, exchange of experiences, psychological support, networking
		Inclusion in health treatment	Integrated treatment, support, specialist treatments, lack of knowledge

### Response

Within the secondary level sub-theme "Response", two primary level subthemes were identified: (1) Expression of Emotions and (2) Adaptation.

Family members of people living with Parkinson's disease experienced and reacted similarly to everyday life with their loved ones. They are aware that living with such an individual brings changes they must accept. Some family members of people living with Parkinson's disease already knew about the disease, while others had no prior knowledge before the diagnosis. Expressing the emotions and feelings of these family members helps us understand how they experience the illness of a loved one. Those who were already aware of the disease described feeling scared, desperate, confused, worried, and sad. They experienced a sense of loneliness, helplessness, and distress, knowing the characteristics of Parkinson's disease and the challenges it brings to their loved ones:

"I knew the disease, so maybe I was more scared inside than my husband. In the beginning, I often felt lonely." (Ana)

"Sadness sometimes really hits me if I'm not in a good mood. Mainly because I'm afraid of the future. Of course, there are also beautiful things; we met a lot of good people." (Mirjana)

"At first, a part of me resisted, got upset, and I felt split. It was not easy." (Vlado)

Family members described that their predominant emotions were unpleasant upon learning of the diagnosis. Fear and worry emerged initially, especially when they and their loved ones faced an incurable disease. Feelings of separation, pain, and even denial were inevitable:

"Sometimes I get feelings of helplessness, sadness, anxiety. I try to take care of him as much as I can." (Nina)

"The feelings are unpleasant, especially since no medicine can completely cure the disease". (John)

"I didn't know the disease. At the time of the diagnosis, fear and much worry first appeared, which are still present today." (Milena)

On the other hand, family members also experienced a certain degree of motivation and resilience, particularly after accepting the disease as a part of their lives. Despite the inevitable changes and the need for adaptation, many family members found positive aspects and acknowledged that the disease had taught them valuable lessons. Through this, we can understand how family members often adjust to living with the disease. Unfortunately, they had no other choice:

"There is always something good in everything bad. We met interesting people, acquired new knowledge, found challenges and a new field of work where I can help myself and others." (Mojca)

"I knew the disease well; when I found out that my husband had it, my world collapsed." (Sonja)

"The disease has taught us a lot; we know how important health and well-being are, that they are not taken for granted. We learned to slow down the pace of life." (Anja)

### Change

A secondary-level subtheme Change consists of three primary subthemes: (1) the Role of family members; (2) Patient characteristics; and (3) Family relationships.

Family members reported that their way of life underwent a complete transformation compared to life before the disease, both for their loved ones and themselves. Personal characteristics and family relationships represent the most significant changes in their role. They acknowledge that the disease decelerates life and requires immense patience. Along with the altered lifestyle, certain restrictions and adjustments arise, as well as changes in carrying out specific activities and helping the patient. The way of life also relies on the stage of the disease the patient is in. As the disease progresses, the demand for inevitable changes in daily life increases. All the family members confirmed and emphasized the changes in everyday life. They stated that these changes intensify with the stage of the disease. As the disease progresses, it becomes increasingly complex and challenging to adapt to the lifestyle, affecting the quality of life for both people living with Parkinson's disease and their family members. Family members emphasize the individual's slower performance of activities, which requires patience and constant adaptation or sacrifice. The most significant change compared with life before the disease is the adjustment of daily activities and the overall deceleration of life. As a result, their role also changes, particularly in terms of caring for the sick. Along with the lifestyle change, family members also report alterations in the activities they used to do together or in how the individual now engages in activities differently than before the illness:

"Our life has been changed. It is a constant need to adjust your lifestyle. Many activities that we used to do together, we no longer do today, or we do them differently." (Ana)

"We must be aware that with the disease comes certain limitations and adjustments that you must accept and adjust your activities accordingly. In life with a patient with Parkinson's disease, it also depends a lot on the stage of the disease itself." (Mojca)

There are also significant changes in the characteristics of the person affected by the disease. The main changes identified and mentioned by family members are primarily related to personality, such as being quick to take offence, withdrawn, irritable, listless, disinterested, and in denial of the illness:

"The drugs changed his personality, and as a result, our life changed a lot." (Ana)

"He has become much more sensitive, quickly offended and withdraws into himself." (Mirjana)

"Father has become significantly more sensitive, weak; he leaves most of the work to me, which is tiring." (Vlado)

"The biggest challenge for me is precisely his lack of interest and listlessness. There are days when he runs out of strength even for communication or a basic conversation." (Nina)

They also mentioned the physical changes of the patient living with Parkinson's disease, namely the lack of independence or the inability to take care of oneself, slowing down, and changes in mobility:

"I see that he is trying hard to walk, but his body no longer allows him." (John)

“He is slow, uninterested in things that are not necessary for life. Otherwise; the disease does not worry him too much." (Milena)

We can confirm that the complete change in lifestyle and adaptation of daily activities to the individual's condition also affects family relationships. These relationships can be either positive or negative, with some remaining unchanged. However, we often find that relationships are worse than before the disease occurred:

"My father and I have become even closer, but at the same time, we both know our weak and good sides better. It also affects other relationships." (Vlado)

"Our relations have changed quite a bit. My husband, for example, has to rely on me more than he did before. But I don't think the disease affects relationships with family and friends." (Nina)

“Attitudes have not changed much. Most of our friends have accepted and are helping us to overcome." (Janez)

### Care

The secondary sub-theme Care consists of two primary-level sub-themes: (1) Care and assistance; and (2) Stress and burden.

As the disease progresses, the patient's level of independence diminishes. Family members provide significant assistance and care, including help with personal care, dressing, cooking, getting up, and offering support and encouragement in daily tasks and physical activities:

"The disease is in the initial stage when minimal help is needed, such as buttoning up and encouraging physical activity. With various activities, we try to maintain such a condition that the disease does not progress too quickly." (Mojca)

"Father needs daily help, it is difficult for him to walk, and he can no longer take care of most of the daily tasks." (Vlado)

Some family members have started engaging in activities they did not participate in before the disease, precisely because of the onset of the disease and to slow down its progression:

"We are much more involved in activities that help limit the development of the disease. So, we go to the hills a lot more. More slowly and carefully to avoid injuries. We also engage in sports that we have not played before, such as table tennis." (John)

"I took on more household chores, especially cleaning. I took over shopping, all transport, lunch delivery from the inn, escorts to doctors..." (Primož)

"I help my husband by serving him, making sure he takes his medicine. If necessary, I help him get dressed. I encourage him to walk and exercise." (Milena)

When caring for people living with Parkinson's disease, especially when they are close family members, it is common for caregivers to forget about themselves or neglect their own needs easily. Consequently, this quickly leads to increased stress and various burdens that family members experience daily. However, we found that many family members believe that despite the changed way of life, constant adaptation, and care for their loved ones, they do not forget themselves, or at least they do not feel that they are neglecting themselves:

"Of course, from time to time I am additionally exposed to stress, which in turn I would associate with the fact that I myself have not yet fully adapted his rhythm to mine." (Ana)

"Stress is present every time there is something unpredictable. I'm lucky that I'm healthy, and somehow I can handle it." (Vlado)

"I try to neglect myself as little as possible, I try to take care of myself as much as I can, I don't neglect others." (Nina)

"At the moment, I don't feel like I'm neglecting myself. I only care about us. My husband is slow but still active." (Milena)

"The biggest burden and stress for me is monitoring the deterioration of my condition because I feel helpless." (Anja)

### Support

The sub-theme of the secondary level, called Support, consists of two primary-level sub-themes, namely: (1) Society; and (2) Inclusion in health treatment.

Many family members found it helpful to be involved in associations when dealing with negative feelings and emotions. Through such involvement, both family members and individuals living with Parkinson's disease find the support they often mention. Joining associations also allows networking, socializing, and sharing experiences with other family members of people with Parkinson's disease. In these associations, they also have access to various educational programs, lectures, and seminars:

"To understand the disease, all family members need knowledge, mutual association, and exchange of experiences. That is why the self-help group for family members of patients in the Society is very important. The meetings are intended for lectures and workshops both for patients and their caregivers." (Mojca)

"Until now, information about the disease has been arranged at camps organized by the Society. At one meeting, we had a meeting with only family members, where we exchanged experiences and information." (Primož)

"Working with family members is always welcome. Many activities are already taking place in the Society, where various seminars, activities, meetings, workshops are held..." (Anja)

Family members want more emphasis on education, awareness, socializing, and psychological support. They also recognize the importance of holistic treatment and their involvement in the treatment and care process. They assist people living with Parkinson's disease, providing daily encouragement and offering psychological support. However, they believe they cannot fulfil these roles to the best of their ability if they are not physically and mentally healthy. Therefore, they believe including family members in the treatment process is crucial, leading to a holistic approach that addresses the needs of people living with Parkinson's disease and their family members:

"I think that family members are given too little time and emphasis in treating the patient." (Ana)

"It takes a lot of work with the individual's family members. Lectures, various workshops, mutual gatherings and exchange of experiences make sense." (Mirjana)

"I lack a lot of knowledge. I would be happy to receive instructions for specific situations that I am experiencing with my father.

## Discussion

This study aimed to explore the perceptions of family members regarding everyday caregiving for a family member living with Parkinson's disease and to describe their role in care and everyday life.

We discovered that family members share a similar response when a loved one is diagnosed with Parkinson's, as they commonly experience discomfort, fear, and worry upon learning that the disease is incurable. Additionally, feelings of separation, pain, and denial frequently manifest. Dekawaty, et al. [19] also found that family members of individuals living with Parkinson's disease most often report sadness, anxiety, inconvenience, hopelessness and fatigue. We found that family members report about adapting to daily living, accepting help, gaining new knowledge, and coping with difficult situations. Others also found that caregivers consistently adjust to cope with the disease's unpredictable nature and the patient's changing needs [8]. Consistent with our research, family members agree that their role changes and accepting the disease as a part of everyday life is necessary. This acceptance enables the creation of a positive and high-quality life for the family and the loved one [23]. Similar findings were also reported by Dekawaty, et al. [19], highlighting the most significant change in adjusting various life activities, cultivating patience, and embracing a slower pace, which can also be viewed as a positive transformation. Ambrosio et al. [22] note that a lot of sacrifice, change and adaptation is required. Life is no longer the same as before the disease; it is also difficult to accept that a loved one is no longer capable of the same activities as before and that their physical ability and personality changes. Our study confirmed family members underwent significant changes in their daily lives due to the illness, requiring increased adaptation in their activities. Lennaerts-Kats, et al. [30] describe that the role of the family members changes the most when diagnosed with Parkinson's disease. They found that a large part of the support of family members relates to the daily help of the loved one. The reasons why family members take on the role of "caregiver" include the increasing dependence of the individuals living with Parkinson’s disease and the desire to provide the loved one with the greatest possible well-being and dignity. They state that they feel compelled to accept these roles as if they have nothing else left. Many seem to view caregiving as a spousal duty and have adapted their lives to it [30]. The change is related to an individual’s characteristics. Physical and psychological changes are highlighted. Perception of changes become more intense when family member develop physical and/or mental limitations [16]. Changes also affect family member relationships in terms of social isolation, marriage problems, and deterioration in interpersonal relationships in general. Decreased socialisation was found to be an important issue also by Perepezko, et al. [31]. However, sometimes there are no changes in family relationship quality, or they even improve, as Perepezko, et al. [31] noted.

In terms of caregiving, family members most frequently reported helping with everyday tasks such as dressing, washing, buttoning, meal preparation, shopping, driving, and managing regular visits to the doctor, as well as involving the patient in medical treatment. Bhasin and Bharadwaj [8] also emphasise that most caregiving involves assistance with everyday tasks. Sturm, et al. [25] note that family members gradually provided their loved ones with more physical, social and emotional support as the disease progressed. Tasks ranged from assisting with daily activities such as bathing, dressing, transferring, cooking, feeding, managing medications, and making financial decisions [25, 32]. Caring for the family member is also evident among non-dependent family members. The family member's well-being becomes a significant concern, and they often accompany them on walks and provide supervision for personal care [22].

We found that family members face daily stress and burdens specifically due to assuming specific responsibilities and providing assistance to the sick individual. Family members state that they are occasionally exposed to additional stress, which they attribute to their ongoing process of adapting to their changed lifestyle. They confirm the presence of stress, particularly when faced with unpredictable events. Family members assert that they try to prioritize self-care despite the added stress. They recognize that taking care of themselves is essential for providing the best possible care to their loved ones. Similarly, Fox, et al. emphasize the importance of recognizing the significant individual differences in response to stressors, such as a loved one's life-limiting illness, distressing symptoms, and assuming the role of a caregiver. Not all family members experience depression, sadness, or burden. This may reflect personality resilience whereby some families adapt and cope better than others [33]. On the other hand, the reduction in social interactions of family members is notably exacerbated by their inability to leave these individuals unattended at home due to safety issues [26]. Many family members in our study believed that the patient's well-being depends on active participation in holistic care and cooperation with specialists and the healthcare team. They also emphasized the importance of ongoing education for family members and patients, emphasizing the value of workshops to exchange experiences, opinions, questions, and advice. Lennaerts-Katst, et al. [30], state that family members emphasize the crucial role of health professionals, who should be mindful that individuals with advanced Parkinson's disease rely on others for basic activities of daily living. Due to apathy or cognitive impairment, individuals are often unable to express what they need. As a result, many individuals feel a greater need for support [30]. Our study supports these findings, as most family members agree they do not receive enough time and attention in the treatment and care process. They emphasize the importance of working together with healthcare professionals. Family members find lectures, workshops, socializing, and sharing experiences in societies highly valuable, as they still have much to learn.

### Strengths and limitations

This study provides an overview of the situation in Slovenia and may be applicable in other countries with healthcare systems that are comparable to Slovenia's. However, the study was restricted to members of society, and perceptions might differ compared to family members not included in social support groups. The duration of living with an individual with Parkinson's disease and the stage of the disease may also influence variations in perceptions among family members.

#### The implication for nursing practice

The results of our study have significant implications for nursing practice. When family members observe changes in the health status of their loved ones, healthcare providers must establish criteria for emotional support and the provision of information. Healthcare teams need to have knowledge and awareness of the coping mechanisms employed by family members in response to the diagnosis and progression of the disease. Collaboration among healthcare teams, relevant associations, and societies is crucial in promoting and guiding the provision of help and support to family members who care for individuals with Parkinson's disease. This partnership can effectively reduce the emotional and physical burden on families responsible for the care of their loved ones with Parkinson's disease.

It is essential to explore the most effective and supportive approaches for family members to provide care for individuals living with Parkinson's disease at home for as long as possible. Our findings indicate that family members desire to actively participate in treating and caring for their loved ones, emphasizing the need for healthcare teams to recognize their important role, especially right after diagnosis. Moreover, determining the optimal timing for transitioning an individual with Parkinson's disease to a nursing home when family members can no longer provide care is also crucial.

## Conclusions

Living with Parkinson's disease is a life full of challenges for the family members. Our study revealed that most family members similarly perceive their experiences. They often experience emotions, and feelings of fear, sadness and despair are intertwined. However, as they come to accept the disease as a part of their everyday lives, they learn to adapt and coexist with it. They also recognize the valuable lessons that the disease has taught them. They agree the key is to adapt and embrace the changes that the disease brings. Both their own and the individual’s daily lives adopt a slower pace. The active involvement of family members in the overall treatment and care of their loved ones is essential. Providing diverse educational opportunities, workshops, social interactions, self-help groups, and platforms for sharing experiences can greatly support individuals who are impacted by the disease. Supporting and empowering family members makes it easier for them to care for their loved ones, leading to an enhanced quality of life for all involved.

### Supplementary Information

**Additional file 1:** **Suppl. 1.** Overview of interview questions.

## Data Availability

The study's datasets cannot be shared to safeguard participants' anonymity, confidentiality, and sensitive input. While the corresponding author has the data, it was generated and analyzed solely for this study. Nonetheless, those interested can request the data from the corresponding author in line with participants' consent forms.
