# Supplementary material for: Family members’ experiences of everyday caregiving for a family member living with Parkinson’s disease: a qualitative thematic analysis study
Source: BMC Nurs. 2024 Feb 6;23:98. doi: 10.1186/s12912-024-01767-6 (PMC10845758; doi:10.1186/s12912-024-01767-6)
Supplement: Supplementary file 1 — Additional file 1: Suppl. 1. Overview of interview questions. [file 12912_2024_1767_MOESM1_ESM.docx]

# Suppl. 1: Overview of interview questions

1. Briefly describe yourself, identify your sex and age, and what is your relationship to the person with the disease?
2. How would you describe life with a Parkinson's patient?
3. What feelings did you experience when you found out that your loved one had Parkinson's disease? Were you familiar with the disease?
4. In what ways do you help your lovedone in everyday life? What is the biggest challenge/concern for you and how does it make you feel?
5. What is the biggest burden for you in your life with a Parkinson's patient?
6. How has your relationship with the patient changed compared to before the illness?
7. Where do you notice the most significant changes?
8. How does the disease impact your relationships with other family members and friends?.
9. Has living with such a patient given you any positive/good things or experiences, if so which ones? What have you learnt from this?
10. Do you think that more emphasis should be given to the relatives in the management and during the course of the illness? If yes, in what ways do you think it would make the most sense?
